# Innovations in Trauma-Informed Care: Building the Nation’s First System of Trauma-Informed Recreation Centers

**DOI:** 10.3390/bs13050394

**Published:** 2023-05-09

**Authors:** Megan R. Holmes, Jennifer A. King, Emily K. Miller, Dakota L. King-White, Amy E. Korsch-Williams, Erica M. Johnson, Tomeika S. Oliver, Ivan T. Conard

**Affiliations:** 1 Center on Trauma and Adversity, Mandel School of Applied Social Sciences, Case Western Reserve University, Cleveland, OH 44106, USA; jak292@case.edu (J.A.K.); ekm40@case.edu (E.K.M.); d.l.king19@csuohio.edu (D.L.K.-W.); axk130@case.edu (A.E.K.-W.); eej13@case.edu (E.M.J.); txd163@case.edu (T.S.O.); ixc130@case.edu (I.T.C.); 2 Levin College of Public Affairs and Education, Cleveland State University, Cleveland, OH 44155, USA; 3 Weatherhead School of Management, Case Western Reserve University, Cleveland, OH 44106, USA

**Keywords:** trauma-informed care, parks and recreation, adverse childhood experiences, systems change, organizational-culture, community, trauma-informed models, leadership development, intervention, organizations

## Abstract

Exposure to adversity and traumatic events affects well-being across important domains of functioning, including mental, physical, social, emotional, spiritual, and neurobiological. Situated as a focal point throughout neighborhoods, recreation centers are a prime opportunity to cultivate spaces of safety and healing. However, current models of trauma-informed care largely do not map neatly onto the recreation organizational structure and functioning. This paper describes the efforts over the past five years to transform the City of Cleveland, Ohio’s 22 recreation centers into trauma-informed Neighborhood Resource and Recreation Centers (NRRCs)––places where children, youth, and adults can readily acquire the support and services they need in an environment in which trauma-informed care principles are fully embedded in the fabric of the organization’s culture. Phase 1 included transitioning the recreation centers to NRRCs, hiring of trained social workers and counselors to work within the recreation centers, and training all recreation staff about trauma. Phase 2 included development of NRRC trauma-informed standards, development of the Trauma-Informed Progress Tool to track change over time, development of Trauma-Informed Leadership Competencies for Center Managers, and ongoing training for the social workers and counselors. We discuss ideas for future work and lessons learned from each phase.

## 1. Introduction

In 2018, the City of Cleveland Mayor Frank Jackson, his administration, and City Council worked tirelessly to pass legislation to treat crime and violence as a public health problem [1]. The rationale presented to City Council was that children and youth who are exposed to ongoing trauma and stress such as poverty, crime, and violence can suffer consequences that can lead to more violence. The aim of the legislation was to address trauma in youth by targeting the city’s 22 recreation centers as places of potential support, healing, and holistic care for local families and the broader community. This pivotal legislation positioned the City of Cleveland to create the first system of recreation centers to implement trauma-informed standards of practice in the United States. Below we describe the work conducted over the past five years, including lessons learned from each phase.

### 1.1. Impact of Childhood Trauma

Exposure to trauma has long been known to affect well-being across important domains of functioning. A trauma is an event, or series of events, or circumstances that are experienced as emotionally or physically harmful and has an enduring impact on someone mentally, emotionally, physically, socially, and/or spiritually, and neurobiologically [2]. In the 1990s, the Adverse Childhood Experiences (ACE) Study illuminated the profound impacts of some forms of early life trauma on lifetime mental and physical health, demonstrating a dose–response relationship between experiences of abuse, neglect, and household dysfunction during childhood, and risk of physical, mental, and behavioral health challenges later in life [3,4]. While the ACE Study was groundbreaking and has since influenced policy, research, and service delivery, these findings were limited because the dose–response relationship demonstrated only correlational associations among individual and family-level experiences and negative outcomes, and important neighborhood and community-level factors were not considered. In fact, research that examines other forms of traumas shows that more than 70% of people in the United States have been exposed to at least one traumatic event [5]. Since the original ACE Study, research that explored important neighborhood and community-level trauma has found that factors such as community violence, lack of neighborhood safety, and discrimination in childhood also influence health outcomes in adulthood. Research has further shown disparities among race, age, socioeconomic, and sexual and gender identities in terms of numbers [6] and types [7,8] of ACEs and other traumas reported. These disparities stem from structural inequalities that shape environmental circumstances in which families and individuals are at greater risk for traumatic experiences. Research has also pointed to the synergetic or compounding effects of experiencing multiple traumas; this is true for individual/household-level traumas––such as those examined in the ACE Study––in combination with community-level traumas [9,10]. For example, Lanier et al. [9] found children who were exposed to mental illness and poverty together had a higher risk of having a special healthcare need, a risk that was higher than for children who had the highest number of ACEs overall. Further, experiencing ACEs or other traumatic events has been shown to have neurobiological impacts on the brain, especially during early childhood, that may have negative effects throughout the lifespan (see [11] for a comprehensive review). In response to the pervasive impacts of childhood trauma, many organizations and systems have moved toward adopting models of trauma-informed care which aim to modify organizational policies and practices to better facilitate healing [12].

### 1.2. City of Cleveland and the Neighborhood Resource and Recreation Centers

The population of Cleveland, Ohio, faces high rates of trauma exposure, rooted in socioeconomic and racial inequalities. Approximately one-third (32.7%) of Cleveland residents live below the poverty line and 46.1% of children live in poverty [13]. Cleveland residents also face high rates of unemployment, low insurance coverage, and low levels of educational attainment. In 2019, the national benchmark for unemployment was 3.2%, whereas unemployment in Cleveland was 13.9% [13]. These inequalities are associated with traumatic events, such as high rates of homicide and violent crime [14], child maltreatment, and food insecurity [15], with a disproportionate impact on Black and Hispanic residents. These statistics underscore the reasons why residents of marginalized communities are at increased risk for mental illness, chronic disease, higher mortality, and lower life expectancy [16] and need more effective, comprehensive resources that help promote healing across all domains of wellbeing.

In Cleveland, recreation centers are situated as focal points throughout neighborhoods and offer an opportunity to cultivate spaces of safety and healing. Recreation centers serve as a major resource to communities across the United States, providing fitness centers and aquatic facilities, healthy living classes, after-school opportunities for youth, programming for older adults, access to computers and the internet, health and wellness programs, and food to community members [17]. The Cleveland Division of Recreation operates 22 recreation centers that offer services and programming at no cost to patrons. Given the accessibility of the recreation centers and the many programs and services already being offered, the Cleveland Division of Recreation recognized that these centers had potential to provide even greater resources to the community. In 2018, the Cleveland Division of Recreation took initial steps toward expanding the recreation centers into trauma-informed environments to reduce the impact of trauma and promote healing and resilience in youth. However, current models of trauma-informed care largely do not map neatly onto the recreation organizational structure and functioning.

### 1.3. Limitations of Current Trauma-Informed Models and Approaches for Recreation Centers

The trauma-informed approach involves creating a safe and supportive environment that empowers individuals to engage in their own healing process, rather than re-traumatizing them. Trauma-informed care emphasizes the importance of understanding the root causes of trauma and uses a strengths-based approach to promote resilience and recovery. However, previous initiatives within youth-serving organizations have largely consisted of training and have yet to integrate a trauma-informed care model with the National Park and Recreation Association’s standards of practice [18]. Additionally, a pure training model may not be sufficient, as the content is mainly designed for clinicians and practitioners such as clinical therapists, social workers, behavioral health specialists, and primary care physicians who work with children and adults as “clients” or “patients.” For example, the widely influential Sanctuary Model was originally developed for use in acute or short-term adult inpatient psychiatric settings [19]. Since its conception, the Sanctuary Model has been applied to many settings, such as domestic violence shelters, drug and alcohol treatment centers, and residential treatment centers, among others [20]. Similarly, the Creating Cultures of Trauma-Informed Care (CCTIC) model, which emphasizes organizational-level culture change, is primarily designed for change in human service organizations such as mental and behavioral health agencies [21,22]. Previous research has acknowledged the importance of a community participatory process and a social–ecological perspective to strengthen trauma-informed practices at multiple levels within communities [23]. Some trauma-informed care models originally developed for health and human service settings have been adapted specifically for school settings, such as the Sanctuary Model [20]. Many school-specific models have also been developed, such as Trauma-Informed Schools for Children in K-12 framework [24], the Trauma-Informed Programs and Practices for Schools [25], and the Trauma-Informed, Resilience-Oriented Schools Toolkit [26], among many others.

Despite goals to transform the recreation centers into trauma-informed environments, existing models of trauma-informed approaches do not translate well to recreation center environments. As such, prior trauma-informed training for staff in youth-based after-school programs was found to be ineffective due to the inability of employees to manage personal trauma along with that of the patrons’, lack of continued funding, as well as content knowledge loss over time [27]. Recreation centers’ primary function is to promote health and wellness through providing many opportunities for physical activity, out-of-school programming, arts and crafts classes, food and meals, and connecting community members [17]. They were not originally intended to provide direct services to individuals seeking mental health or behavioral health intervention or treatment, which is the assumption underlying, and driving, many trauma-informed care models. Further, recreation centers are not designed to provide academic education, like schools, and thus the interactions and relations among staff and patrons are not comparable. In addition, the organizational structure of health and human service agencies, therapeutic organizations, and schools are vastly different from recreation centers, which are typically embedded within the larger structure of city governments. Furthermore, recreation centers in the United States are guided by organizations such as the City Park Alliance, Urban Land Institute, Trust for Public Land, or the National Recreation and Park Association (NRPA). The NPRA is, however, the only organization that offers nationally recognized accreditation.

The NRPA’s Commission for Accreditation of Park and Recreation Agencies (CAPRA) is the national accreditation body that outlines metrics for parks and recreation centers to provide high-quality services to communities. Becoming accredited by the CAPRA helps recreation centers uphold the highest standards of services within communities. The CAPRA guidelines, originally created in 1989, encompass a total of 154 standards that span across ten content areas, which are (1) agency, (2) planning, (3) organization and administration, (4) human resources, (5) fiscal management, (6) programs and services management, (7) facility and land use management, (8) public safety, (9) risk management, and (10) evaluation, assessment, and research. The CAPRA model is designed to enhance parks and recreation centers’ overall services to meet or exceed national established benchmarks at the local or regional levels. In order to become accredited, a recreation center needs to provide evidence that their respected agency has met an initial 142 CAPRA standards within their first accreditation period. Currently in the United States, there are nearly 170 park and recreation agencies from 38 states that have achieved this accreditation.

While the CAPRA provides the standards for creating high-quality recreation centers, they do not provide guidance on how to make recreation centers a place of support, healing, and holistic care for communities who experience high levels of traumatic events. Similarly, the current trauma-informed models that focus on organizational change fail to incorporate aspects of systems change that are necessary for recreation centers. The Substance Use and Mental Health Services Administration’s [2] trauma-informed principles of safety; trustworthiness and transparency; peer support; collaboration and mutuality; empowerment, voice, and choice; and cultural, historical, and gender issues can be applied in non-clinical contexts, but there is no existing model comprehensively applying these principles in the city-wide recreation center context that aligns with the CAPRA standards.

### 1.4. Building the Nation’s First System of Trauma-Informed Recreation Centers

In 2018, the City of Cleveland invested substantial financial resources and undertook a massive effort to provide targeted coordinated programs and supports to address community-level trauma, while bolstering and leveraging already existing strengths within the recreation centers (e.g., relational connection between staff and patrons; free sports and recreation programming; accessibility of the 22 locations spread throughout the City of Cleveland). The ultimate goal was to cultivate spaces in which Cleveland residents could thrive. While the work has spanned nearly 5 years, there are distinct phases and lessons learned from the efforts to transition the City of Cleveland’s recreation centers to the nation’s first system of high-quality trauma-informed Neighborhood Resource and Recreation Centers (NRRCs).

## 2. Phase 1: Establishing a Trauma-Informed Foundation

Phase 1 spanned 2018 to 2019, which included (1) transitioning the City of Cleveland’s 22 recreation centers to NRRCs, (2) contracting with a local non-profit behavioral health organization to hire trained social workers and counselors to work within the recreation centers, and (3) training all recreation center staff about exposure to trauma and its lasting effects. This phase was led by a local non-profit behavioral health organization who collaborated with the authors in developing and co-facilitating the training as well as conducting the evaluation.

### 2.1. Recreation Centers to Neighborhood Resource and Recreation Centers

Given that the recreation centers are an essential resource embedded within Cleveland neighborhoods where thousands of children, youth, adult, and senior residents visit annually, the centers were identified as the prime system to extend resources to community members. Transitioning the recreation centers to NRRCs meant that programming was extended beyond traditional sports and recreation to include activities and programs aimed at addressing social determinants of health, which are the social and economic factors such as poverty, education, housing, discrimination, and access to healthcare that can have a significant impact on an individual’s health outcomes and overall well-being. Expanded programming centers around the following six areas:

1. Youth and adult education, ranging from K-12 enrichment programs to post-education skills training.
2. Job and career readiness, including career planning, job training, and job-placement services for adults.
3. Health and wellness education, raising awareness of both physical and emotional issues, to aid people of all ages.
4. Youth leadership and development, including use of mentors who grew up in situations similar to what youth face today and can serve as role models.
5. Arts programming, both for performing and visual arts.
6. Sports and recreation, including both traditional recreation sports and nontraditional ones such as fencing and rowing.

The expansion of programs allowed for the NRRCs to begin to offer holistic care and resources for community members.

### 2.2. Training All Recreation Center Staff

Staff training on trauma-informed care is a key element, and often the first step, in trauma-informed organizational transformation [2]. Research has established that participation in trauma-informed training can significantly improve staff knowledge, attitudes, and behaviors related to trauma-informed practice [28]. While many training efforts focus on social service and educational sectors, one governmental effort of note is Baltimore’s citywide trauma-informed training initiative for all government employees. The training initiative positively impacted both organizational factors such as climate, morale, and managerial support as well as individual factors such as deriving pleasure from doing one’s work well [18]. 

During the first three months of the transformation effort, a series of 3 h professional development sessions were held on topics including an introduction to trauma-informed care, developmental impacts of trauma, signs and symptoms of trauma, conflict mediation, and strategies for de-escalation. Nearly 300 individuals––from recreation center desk staff, coaches, custodians, and security personnel to community partners such as violence interrupter teams––received training. Pre- and post-test evaluations were performed for each training session and showed an increase in knowledge. Each session also included space for City employees to reflect on what impact the previous training has had on their work. Some staff shared that they were utilizing aspects of trauma-informed care in how they treat youth and children; the training further benefited the staff by giving them a shared language and boosting positive energy and pride. Other recreation staff stated that while they knew the children they served had significant challenges in their lives, they had not known how to react or respond to the behaviors they saw resulting from those challenges. The trauma-informed care framework offered them information to better understand that the behaviors of these youth may be linked to traumatic experiences and provided techniques for de-escalation. Staff reported that they noticed that their own shifts in behavior changed the outcomes of youth each day.

### 2.3. Hiring Trained Social Workers and Counselors

An essential component of the Phase 1 transformation effort was the decision to staff all of the NRRCs with trained social workers or counselors to provide short-term direct services such as case management, crisis intervention, psychoeducational support groups, and resource referral to residents in order to assist them in accessing the resources and supports they need. These bachelor’s or master’s level providers, initially given the title Trauma Coaches, were employed by a local non-profit behavioral health organization. The Trauma Coaches split their time between two to three NRRCs each, while also providing services in K-12 schools and in the community as needed. After the completion of the training series, the Trauma Coaches acted as stewards of the trauma-informed initiative, holding monthly professional development sessions for the staff at their respective NRRCs. The objective of these sessions was to reinforce the messages from the initial training and introduce a variety of topics to expand the understanding of developing a trauma-informed system for youth and adults who visit the NRRCs.

### 2.4. Phase 1 Lessons Learned

Given the innovative nature of transforming recreation centers into high-quality, trauma-informed NRRCs, there were bound to be lessons learned from the early phase of this work. Over the course of Phase 1, the City leaders from the Division of Recreation and the Office of Prevention, Intervention, and Opportunity for Youth and Young Adults met nearly weekly to discuss the ongoing work and identify ways to make improvements in the next phase. Through these discussions, there were key areas that were identified. It became apparent that training about trauma was not enough to become trauma-informed and that there needed to be a stronger focus on organizational cultural changes within the system. There also needed to be a focus on elevating the quality of the NRRCs, which needed to happen simultaneously as the centers worked towards becoming trauma-informed. City leaders also acknowledged the importance of sustainability of the Trauma Coaches. The current model meant that the Trauma Coaches were employed through the local non-profit behavioral health organization and then contracted by the City to work year-to-year. It was determined that a better model was to have the Trauma Coaches employed by the City in order to support the sustainability of the program.

Reflecting back over the past five years, we identified additional lessons learned from Phase 1 that could be useful recommendations for other cities that may be considering transforming their recreation centers.

- Adequate time for strategic planning between legislation passing and program starting should be built into the first year of the program. Strategic planning should at minimum include a 5-year plan with mapped-out goals for each year of the programs.
- Measuring organizational readiness for change and providing support in this area is recommended.
- Rather than training all recreation staff at the same time, training should be conducted in stages starting with city leadership, followed by Center Managers, and then the remaining recreation staff.
- Tools, resources, and strategies should be available to staff early on to address secondary trauma experienced by staff, particularly for recreation systems located within cities with high levels of violence and crime.
- The city should adopt a trauma-informed city-level ordinance so that all departments and divisions have a shared understanding and language around trauma.

## 3. Phase 2: Organizational Change

While Phase 2 was intended to start in 2020, the COVID-19 pandemic delayed the continuation of the project until 2021. During the first months of the pandemic, the NRRCs closed down and were subsequently utilized as COVID-19 testing sites and later as vaccination centers. Some NRRCs opened during the period when schools were closed and offered space for children to do their online learning. When the trauma-informed program resumed, Phase 2 included (1) development of NRRC trauma-informed standards, (2) development of a progress tool to track change over time, (3) development of trauma-informed leadership competencies for Center Managers, and (4) ongoing training for the Trauma Coaches. Phase 2 was led by the authors who developed trauma-informed standards, the progress tool, leadership competency, and provided the ongoing training.

### 3.1. Development of the NRRC Trauma-Informed Standards

Phase 2 of the NRRC organizational change effort began with the development of NRRC Trauma-Informed Standards, which are guiding principles in which the recreation center policy and practice across all levels of leadership and staff should operate. These standards were created by converging the CAPRA Standards (see Section 1.3) and SAMHSA’s ten implementation domains for a trauma-informed approach [2]. The ten trauma-informed implementation domains for organizations include (1) governance and leadership; (2) policy; (3) physical environment; (4) engagement and involvement; (5) cross-sector collaboration; (6) screening, assessment, and treatment services; (7) training and workforce development; (8) progress monitoring and quality assurance; (9) financing; and (10) evaluation [2]. Previous research has found that inclusion of the ten domains during change management interventions has often resulted in greater support from leadership and staff of the new initiatives and more streamlined process implementation [29]. In addition, SAMHSA’s six key principles in generating trauma-informed care were embedded throughout the Standards (safety; trustworthiness and transparency; peer support; collaboration and mutuality; empowerment, voice, and choice; and cultural, historical, and gender issues).

**Figure 1:**
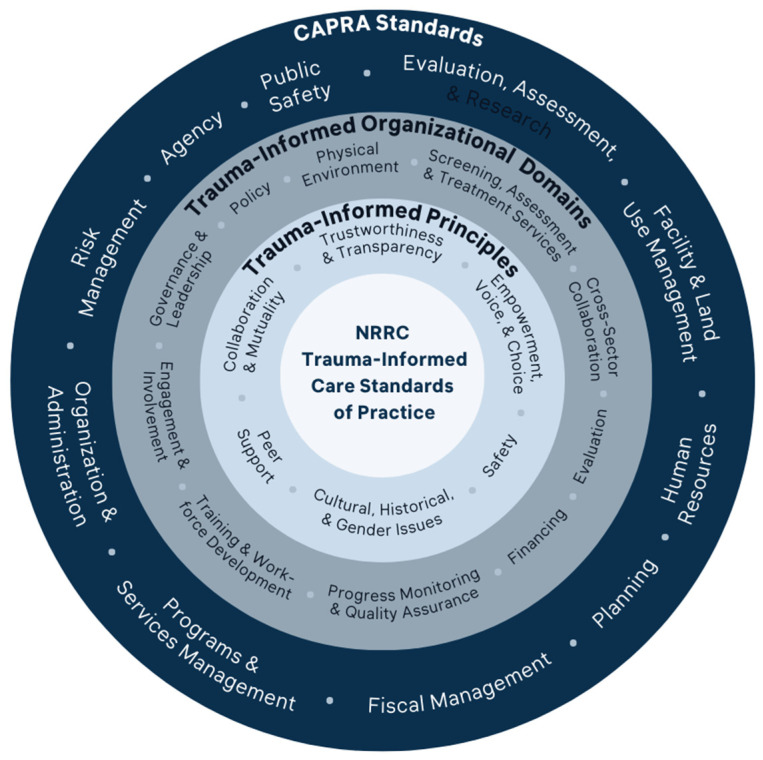
Circumplex of the trauma-informed standards for the NRRCs.

The development of the NRRC Trauma-Informed Standards began with organizing CAPRA standards into congruous SAMHSA trauma-informed organizational implementation domains. This approach allowed us to develop a framework that attended to both trauma-informed practice and CAPRA’s recommended best practices. Figure 1 illustrates the development of the trauma-informed standards for the NRRCs. From outside inwards, the outermost ring represents the CAPRA standards, the next ring represents SAMHSA’s trauma-informed organizational implementation domains, followed by the last inner ring that represents SAMHSA’s trauma-informed principles.

#### 3.1.1. Benchmarking

Prior to integrating SAMHSA principles and CAPRA standards, we benchmarked other metropolitan parks and recreational facilities with patrons of similar demographics. Benchmarking was important to further align NRRC’s trauma-informed initiatives with those of high-performing parks and recreation facilities as well as to survey challenges faced by similar organizations in integrating trauma-informed approaches with operational and regulatory (regional and national) demands. However, importantly, we found that no other parks and recreation system to our knowledge in the country was engaging in as comprehensive of an effort as the Cleveland NRRCs.

We further took into account the level of community violence in the neighborhoods in which the NRRCs are located. Given that this organizational change effort is trauma-informed, we considered how different forms of trauma exposure may be incorporated into the NRRC Standards and accordingly into organizational learning and staff awareness. For instance, a recreation center situated in a neighborhood with higher crime rates may be stronger within the CAPRA standard for public safety, law enforcement, and security.

#### 3.1.2. Manager and Staff Feedback

In addition to benchmarking, it was also critical to gain input on the proposed trauma-informed standards from NRRC managers and staff, including Regional Managers, Center Managers, and program staff. We utilized a change management approach, where research has shown that practicing inclusion during change management processes helps to solidify commitment to the change [30]. It also communicates their level of involvement in the change process and any redesign of their job roles to accommodate new policies, procedures, and practices. We held four focus groups with staff and managers who acted as “trauma-informed champions” to gain a better understanding of the opportunities and challenges within the NRRCs in supporting the standards. Questions centered on the NRRC’s capacity to build a trauma-informed culture by aligning with the SAMHSA’s organizational domains. We explored the strengths and barriers in day-to-day operations, training, and accountability metrics. Such strengths and barriers to success were noted to be embedded within the roles of the staff and managers, NRRC management and City leadership, organizational systems, and government regulations. At the conclusion of the feedback sessions, staff members and managers described feeling a greater sense of ownership within the change management process.

#### 3.1.3. Patron Feedback

We additionally sought feedback from patrons of the NRRCs to explore the recreation facilities that provided a safe and supportive environment. Three sessions were held consisting of youth patrons, caregivers of youth patrons, and adult patrons. Feedback revolved around policies and practices that promote physical and psychological safety, transparency in communication on program offerings, as well as interactions with staff. Findings among patrons were then utilized to support the development of the standards.

#### 3.1.4. Leadership Team Feedback

One feedback session was held with the NRRC leadership team to further understand the organization’s governance and leadership competencies, financing structure, progress monitoring and quality assurance of its systems, and engagement and involvement of staff and patrons. Feedback regarding the organizational dynamics aided us in refining the strategic framework to incorporate trauma-informed practices within the NRRCs overarching systems to enhance staff development and patron care.

### 3.2. Development of TI-NRRC Progress Tool

Building from the foundation of the NRRC Trauma-Informed Standards, next we developed the Trauma-Informed NRRC (TI-NRRC) Progress Tool, which included indicators that measured progress towards becoming a high-quality, trauma-informed NRRC. The purpose of the TI-NRRC Progress Tool is to identify strengths as well as opportunities for improvement in each of the NRRCs. The Tool was designed to be a collaborative assessment process, offering an opportunity for managers at each of the NRRCs to reflect on their center’s process toward becoming a high-quality, trauma-informed resource for the Cleveland community.

#### 3.2.1. Development of Measurable Indicators

Within each of SAMHSA’s 10 organizational implementation domains, we developed potential indicators informed by both SAMHSA’s trauma-informed principles and the CAPRA Standards. First, we reviewed trauma-informed organizational assessments such as the Trauma-Informed Practice Scales [31], Creating Cultures of Trauma-Informed Care (CCTIC) Self-Assessment [21], Standards of Practice for Trauma-Informed Care in Oregon [32], and the Trauma-Informed Care in Behavioral Health Services protocol [2], among others. Despite there being a number of trauma-informed organizational assessments, none to our knowledge sufficiently captured organizational and functional elements of a recreation center. Rather, we utilized these tools to identify constructs of trauma-informed care relevant for the NRRCs and then developed trauma-informed indicators where managers could report on a five-point scale ranging from “Not yet implemented” to “Exemplary/Exceeds standards” to evaluate the extent to which the domain items and indicators have been completed by their NRRC. We then mapped the 154 CAPRA Standards onto the SAMHSA’s ten organizational domains. Each CAPRA Standard provides suggested evidence for compliance, which we utilized to develop standard indicators and utilized the same scale as the trauma-informed indicators. This approach allowed us to merge trauma-informed care with the CAPRA standards.

#### 3.2.2. Managers and Leadership Feedback

As with the previous stage of the Standards development, it was critical to gain input on the proposed indicators from NRRC managers and leadership. This ensured that the language and framing of each indicator was relevant to recreation and accurately reflected the scope of work conducted within the centers. During early rounds of feedback, it became apparent that some of the indicators were outside of the control of the managers, and their ability to make progress on those indicators resided at the leadership level. For example, most written documentation (e.g., common signage in the NRRCs stating the mission, values, or code of conduct; codes, laws, and ordinances pertaining to public safety; and operation policies and procedures) is issued by the administration. Those indicators were flagged as “Division of Recreation/Public Works issues/provides,” but remained as an indicator within the TI-NRRC Progress Tool. It was determined that this method would provide a level of accountability for the leadership team to continue moving the needle toward becoming high-quality, trauma-informed NRRCs.

Feedback by managers and leadership also informed our decision to structure the TI-NRRC Progress Tool into tiers. The original version was lengthy, covering all ten organizational implementation domains with a total of 272 indicators. Jointly with NRRC managers and leadership, we determined that the TI-NRRC Progress Tool needed to support building capacity over time while motivating the NRRC managers to continue making progress. Thus, we reformatted the TI-NRRC Progress Tool to five tiers, where the first tier focuses on foundational items, and then each tier builds on the previous one. Once the threshold for a tier is met, the subsequent tier is added at the next assessment period progressing towards all tiers being included. Because of the tiered approach, it was also determined that the Tool would be implemented quarterly.

#### 3.2.3. Pilot and Baseline Data

The TI-NRRC Progress Tool was piloted to test the methods and procedures. Baseline data were collected across all participating NRRCs. Specifically, the pilot assessed the feasibility of the tool implementation and allowed the NRRCs to determine and provide any necessary revisions to the TI-NRRC Progress Tool protocol. The TI-NRRC Progress Tool was completed collaboratively by the NRRC Regional Managers and Center Managers as intended for Tier 1, and after each domain section, the NRRC Regional Managers and Center Managers assessed the feasibility of the domain section through a series of written reflection questions. The baseline data for Tier 1 of the TI-NRRC Progress Tool was scored and assessed for expected responses and variance. The additional pilot questions were analyzed for feasibility themes, barriers, and facilitators. Results of the pilot indicated that the TI-NRRC Progress Tool functioned as intended. Center Managers reported that completing the TI-NRRC Progress Tool helped them to think through current NRRC operations and opportunities for improvement in a constructive, comprehensive, and systematic way. The TI-NRRC Progress Tool also served as a useful guide for facilitating conversations about the NRRC’s strengths, challenges, and areas for growth. Importantly, Center Managers expressed hope that the TI-NRRC Progress Tool will continue to be implemented in the future, highlighting the value and utility of the Tool for making systemic changes.

#### 3.2.4. Progress Tool Report and Improvement Plan

Baseline results were compiled into a TI-NRRC Progress Tool Report for each NRRC that showed the current assessment. Future assessments will compare the current assessment to the previous period, allowing for a high-level view of progress made over time. The TI-NRRC Progress Tool Report is provided to the Center Manager and Regional Manager ahead of an improvement plan meeting where they discuss the results of the TI-NRRC Progress Tool and develop a plan for improvement over the subsequent 90 days. While the NRRCs have quarterly improvement plans, it is not expected that the NRRC will progress through a tier each quarter. Rather, the goal is to show continuous improvement overtime.

### 3.3. Development of Leadership Competencies

To further support progress toward meaningful and sustainable trauma-informed systems change, we developed the TI-NRRC Leadership Competencies Framework to complement the Progress Tool and support the development of trauma-informed leadership skills for NRRC managers. Similar to the other components of the effort, these competencies integrated a trauma-informed approach with relevant NRPA standards and guidelines. Specifically, the Leadership Competencies Framework drew on Certified Park and Recreation Professional (CPRP) and Executive (CPRE) certification requirements, which encompass important leadership responsibilities and skills in the field of parks and recreation, such as communicating effectively, facilitating programming, managing finances, attending to human resources processes, and overseeing the operations of NRRCs. Similar to the CAPRA standards for recreation centers, CPRP and CPRE certification is a formal accreditation governed by NRPA and the National Certification Board (NCB). One aim of integrating these standards into the Leadership Framework was to help prepare managers for success if they wished to pursue certification formally. Another key goal of incorporating CPRP/CPRE certification standards into the Leadership Framework was to further develop the capacity for high-quality management of recreation centers, ultimately supporting the vision for a high-quality system of NRRCs. However, much like the CAPRA standards, the CPRP and CPRE standards do not naturally align with a trauma-informed approach.

#### 3.3.1. Staff and Leadership Feedback

We began work on the development of the Leadership Framework by conducting a small focus group of managers and staff, with the goal of obtaining their input relative to desired leadership characteristics of managers at various levels, current job expectations, and ways in which trauma-informed principles were currently being enacted within the NRRCs. This feedback was synthesized into themes that informed the process of content development and the eventual structure of the framework. For example, safety—and lack thereof—arose as an important theme that was prioritized for the framework. Themes were also shared with NRRC leadership to support ongoing communication of strengths and areas of concern regarding the experiences of staff and patrons in the NRRCs. A second, larger focus group was conducted after the initial draft of the framework was constructed to provide managers the opportunity to review, discuss, and comment on each aspect of the tool. The insight, feedback, and recommendations provided by the managers was essential to every aspect of this process.

Our vision for the framework was that it would support managers in progressively developing leadership competencies, while also providing opportunities to acknowledge and build upon the many skills they already had in place. Recognizing the complexity and demands of the work managers were engaged in on a daily basis, we were cautious to avoid unintentionally creating additional burdens through this process. Using feedback from the managers and insight we gained throughout our involvement with the project, we determined that designing the tool using a tiered approach to skill building would improve the overall utility and effectiveness of the tool, as well as the experience of the managers in using the framework.

#### 3.3.2. Infusing a Trauma-Informed Perspective

We then merged the specific competencies within each of the five CPRE domains (communication, finance, human resources, operations, and programming) with trauma-informed principles and language. We overlaid the competencies with the six key trauma informed principles (safety; trustworthiness and transparency; collaboration and mutuality; peer support; empowerment, voice, and choice; cultural responsiveness and inclusivity) to ground the framework in a trauma-informed approach. We again condensed, reframed, and re-envisioned many of the original CPRP/CPRE expectations to align with NRRC values, the essential aspects of leadership for NRRC managers, and the core aspects of trauma-informed leadership.

### 3.4. Ongoing Training for NRRC Social Workers and Counselors

In response to the need for sustainability, Phase 2 of the transformation process entailed making the Trauma Coaches internal employees of the City. Rather than being supported by an external agency, the City of Cleveland hired the social workers and counselors and changed their title to Social Support Services Specialists (S4) in an effort to reduce stigma. In order to build community, enhance relational health, and create shared language and knowledge, the S4 team received monthly 3 h professional development sessions based on the Trauma and the Brain Training Series [33]. This case-based curriculum is designed to provide the specialists with foundational knowledge about the sequential development of the brain, the impact of complex child trauma on the brain’s stress response system, and neurodevelopmentally informed approaches to treatment with children and families [33]. In addition, each session included teambuilding and relational health-supporting exercises, processing of successes in working with youth, opportunities for peer support around specific cases, and exploration of the system change process. At the start of each monthly session, S4 team members were encouraged to share “wins” they experienced at their centers. This practice revealed their commitment to and passion for their work, acknowledging the opportunity to launch initiatives such as monthly mental health check-ins with grieving community members post neighborhood violence. Post-training evaluations were conducted, and S4 staff consistently noted they valued learning practical skills to apply directly to their work.

### 3.5. Lessons Learned

Phase 2 of the transformation efforts began in the context of various changes resulting from the onset of the COVID-19 pandemic. The world was changing, and while the recreation centers were able to quickly pivot to providing support around both COVID-19 testing and vaccinations, it was not without significant stress to both NRRC staff and patrons who relied on the centers for support. Furthermore, during this time, there were numerous high-profile incidents of police brutality and racial violence persistently elevated through social media and news outlets. Once the NRRCs re-opened, it was apparent that there was a need to fundamentally change the way services were provided by the city. As such, there is now even more need for culturally affirming mental health supports and programming in community sites such as the NRRCs. The S4 team is currently comprised of 11 staff, 2 supervisors, and 1 director; each staff person splits their time between two recreation centers, while also providing services in local schools and neighborhoods as needed. Expansion of this team, allowing for one S4 per NRRC, would be key in meeting the need for additional mental health support. Furthermore, having an S4 at each NRRC would further build trust within the community by having a consistent presence and access to the patrons.

Phase 2 also occurred during a transition of leadership in the City of Cleveland. The election of Mayor Justin Bibb brought additional changes in top-level leadership that oversaw the trauma-informed NRRC efforts. These transitions highlighted the need for onboarding and ongoing training for city administration to create shared language and understanding around trauma and trauma-informed care. Furthermore, the initial training of the NRRC employees occurred at the beginning of the project in 2018. Phase 2 shifted to creating trauma-informed organizational tools to measure change within the NRRCs. Combined with the pause from the COVID-19 pandemic and the work conducted in Phase 2, with the exception of the S4 team, the NRRC staff did not receive ongoing trauma-informed training. While the creation of the Trauma-Informed Progress Tool and the Trauma-Informed Leadership Competencies are essential for measuring change over time and assisting in long-term change, a missed opportunity was that NRRC staff did not receive ongoing professional development on implementing trauma-informed practices within their work and day-to-day interactions.

## 4. Phase 3: The Future

Building on our progress made from previous phases and incorporating lessons learned from Phases 1 and 2, we have identified essential areas of future work below.

### 4.1. All Staff Professional Development Series on Trauma-Informed Care

While Phase 1 focused on training staff about trauma and trauma-informed care, Phase 2 shifted to creating tools to measure change overtime and supporting the S4 team. The critical role of leadership in fostering trauma-informed organizational change has been well documented [34,35]. Trauma-informed care can be championed by anyone at any level within an organization. However, senior leadership plays a critical role in modeling trauma-informed principles, allocating resources, and changing organizational policies and practices to align with trauma-informed approaches. The transition to the new Mayor during Phase 2 meant transitions in the leadership team overseeing the trauma-informed NRRC project. This highlights the need for leadership to receive ongoing training in trauma-informed care in order to create shared language, understanding, and buy-in. To this end, it is crucial that everyone, from NRRC staff to upper-level City administration, receives ongoing training and professional development in trauma-informed practice and its implementation in the NRRCs. In order to create culturally affirming places of healing, professional development needs to include a focus on racialized trauma and how to combat discrimination and prejudice while promoting equity, justice, and healing for historically and systemically marginalized communities.

We recommend that management and staff members receive ongoing professional development that includes module-based learning accompanied by practical skill application. In partnership with the Neurosequential Network, NRRC Regional Managers, managers, and staff can receive a six-session training series introducing them to the Neurosequential Model of Sport (NMS), which is an evidence-informed model based on the Neurosequential Model of Therapeutics [36]. NMS presents the set of core concepts related to brain organization, the stress response and neuroplasticity applied to the coaching, training, and supporting youth in community settings.

Additionally, the ongoing monthly professional development series with the S4 team should continue, building upon the previous focus on the impact of trauma on brain development and how to integrate the knowledge of the brain and nervous system into their work with children and youth. This training series should also focus on racialized trauma and its impacts. The professional development series should utilize a train-the-trainer format so that, upon completion, the S4 team will be prepared to take leadership in facilitating additional community building and professional development of NRRC managers, staff, and community members.

### 4.2. Expand Trauma-Informed Care Efforts to Involve Youth and Adult Community Members

Engaging youth and families in training and programming focused on trauma-informed care is important because trauma can have significant and generational impacts on health and overall well-being [3,4]. Trauma-informed care involves understanding the impact of trauma on individuals and developing strategies to support their healing and resilience. By training community members on trauma-informed care, they can gain a better understanding of their own trauma-responses and, in turn, improve their relationships and strengthen their families and neighborhoods. Youth have a particularly poignant opportunity to bring about change in their communities. Youth-led peer support and violence prevention programs have been established across the country, incorporating trauma-informed and restorative practices [37,38,39]. By promoting trauma-informed care among youth, youth can be agents of change by being empowered to help create more supportive and safe environments. In turn, this has the potential to create a generation of individuals who are better equipped to navigate the challenges of life with compassion, empathy, and resilience. We recommend utilizing an intergenerational model where both youth and adults are trained about trauma and trauma-informed care practices and involved in the creation of programming such as peer-support community violence prevention in order to facilitate intergenerational healing.

### 4.3. Ongoing TI-NRRC Progress Tool Implementation and Improvement Plan

Based on feedback gathered during a series of focus group discussions and our experiences as we developed the tool, we plan to incorporate the Leadership Competencies into the TI-NRRC Progress Tool, to support a synthesized and more holistic approach to trauma-informed organizational and leadership practices. This approach will reduce potential redundancy in processes and potential burden on managers, streamline procedures and documentation, and incorporate all aspects of organizational and leadership development into one competency building tool. A key aspect will involve support in the implementation of the TI-NRRC Progress Tool, specific to the area of leadership development. Training and consultation will be essential in supporting NRRC managers in progressing beyond the foundational leadership competencies and providing ongoing skill development, managing burnout, and applying trauma-informed leadership principles to their everyday work.

The TI-NRRC Progress Tool should be implemented on a regular basis. We recommend that in addition to the TI-NRRC Progress Tool Report and Improvement Plan for each NRRC, an additional Summary Progress Tool Report be prepared for the City of Cleveland NRRC Administration that shows progress made for each NRRC as well as the overall NRRC department. The Summary Report should include the Progress Tool indicators that are specific to the City of Cleveland NRRC Administration. Following the analysis and dissemination of the Summary Report to the City of Cleveland NRRC Administration, a meeting should occur with the City of Cleveland NRRC Administration to discuss the results of the Summary Report and develop a plan for improvement over the next 90 days. Creating sustainability will be a key aspect of the next phase of work. It will be essential to train City administrators on the TI-NRRC Progress Tool data collection, online survey use, data analysis, report building, and improvement planning sessions.

### 4.4. Trauma-Informed Policy Review

One of the key elements of trauma-informed organizations is resisting re-traumatization of the people served. Quite often this is conducted by daily practices and interactions with patrons. However, policies and procedures developed from a trauma-informed lens must be in place to ensure that the practices of NRRCs are not traumatizing or re-traumatizing to managers, staff, or patrons. In order for the NRRCs to operate from a trauma-informed lens, policies and procedures must align with trauma-informed care principles. Establishing an NRRC mission and vision that embraces a trauma-informed lens is one key starting point for this process. During this next phase, we also recommended that the City of Cleveland create several new policies and procedures in order to meet the CAPRA standards. These new policies and procedures will need to align with trauma-informed care principles of the NRRCs.

## 5. Conclusions

Recreation centers serve as focal points of activity for many youths and their families. While the value of sport and physical activity is clear, more broadly, recreation centers have the potential to serve as community hubs where neighborhood residents can receive comprehensive support and care. However, if systems or organizations want to achieve trauma-informed organizational change, consideration should be given to the fact that sustainable change takes time. Ongoing commitment and effort are required in order to move the needle toward creating and sustaining trauma-informed environments. Expanded intergenerational programming, on-site mental health services, and the integration of trauma-informed practices can help recreation centers to improve outcomes for youth exposed to trauma and build stronger families and stronger neighborhoods. Recognizing these opportunities, cities can implement trauma-informed care within their recreation centers and provide services to children, youth, and adults in order to combat many of the challenges associated with experiencing trauma. Through this work, we have demonstrated that recreation centers can be potential places of support, healing, and holistic care for local families and the broader community.

## Data Availability

Data sharing is not applicable to this article.
